# Assumed Lighting Direction in the Interpretation of Cast Shadows

**DOI:** 10.1177/2041669518790576

**Published:** 2018-07-31

**Authors:** Tomomi Koizumi, Hiroyuki Ito, Shoji Sunaga, Masaki Ogawa, Erika Tomimatsu

**Affiliations:** Graduate School of Design, Kyushu University, Fukuoka, Japan; Faculty of Design, Kyushu University, Fukuoka, Japan; Research Center for Applied Perceptual Science, Kyushu University, Fukuoka, Japan; Faculty of Design, Kyushu University, Fukuoka, Japan; Japan Society for the Promotion of Science, Tokyo, Japan; Research Center for Applied Perceptual Science, Kyushu University, Fukuoka, Japan

**Keywords:** three-dimensional perception, depth, frames of reference, light

## Abstract

Assumed lighting direction in cast-shadow interpretation was investigated. Experiment 1 used an ambiguous object–shadow-matching task to measure bias in shadow-matching direction. The shadow-matching bias was largest when the lighting direction was on average 38.3° left from above (a median of 25.1°). Experiment 2 tested the effect of body posture (head orientation) on cast-shadow interpretation using stimuli aligned in a head-centrically vertical or horizontal orientation. The below-shadow (light-from-above) bias in the head-centric frame was robust across the sitting upright, reclining-on-the-left-side, reclining-on-the-right-side, and supine conditions. A right-shadow (light-from-left) bias in the head-centric frame was found for the sitting upright and reclining-on-the-right-side conditions. In the reclining-on-the-left-side condition, shadow biases to the gravitational *below* direction and head-centric right direction may have cancelled each other out. These results are consistent with findings from previous shape-from-shading studies, suggesting that the same light-source assumption is applied to shading and shadow interpretations.

## Introduction

Shading and cast shadows are known as pictorial depth cues. Such cues allow the observer to recover the three-dimensional structure of the world. Although there has been much research on shading, fewer studies have focused on cast shadows. This study investigated lighting direction assumptions used in the interpretation of ambiguous cast-shadow displays and the frame of reference such assumptions are based on.

Some researchers have explained the ability to perceive shape from shading in terms of the *light-from-above* assumption (Howard, Berstrom, & Ohmi, 1990; Kleffner & Ramachandran, 1992; Ramachandran, 1988a, 1988b), whereas others have proposed the *light-from-above-left* assumption. Sun and Perona (1998) used a visual search paradigm to determine the best shading orientation and showed that the most effective depth effect was acquired when the shading axis was tilted to 23.3° counterclockwise from the top for right-handers (7.9° for left-handers). Mamassian and Goutcher (2001) used a depth-ambiguous figure and measured the transition of the rates of two types of depth appearance by changing the stimulus image orientation. The results showed that the assumed lighting direction was 26.1° tilted counterclockwise from the top. Andrews, Aisenberg, d’Avossa, and Sapir (2013) showed that the lighting direction bias was 29.79° or 8.16° tilted to the left from the top for first-language English or Hebrew participants, respectively, suggesting a cultural habituation effect. Other studies have also found a light-from-above-left bias in shading interpretation; for example, 10.7° (Mamassian & Landy, 2001), 18° (Stone, Kerrigan, & Porrill, 2009), and 22.3° (Gerardin, Kourtzi, & Mamassian, 2010) from the top. Thus, there seems to be a general consensus on the light-from-above assumption in the shape-from-shading paradigm and on the inclination of the preferred lighting direction to the left from the top although there are some variation in the amount and cause of the inclination. The interpretation of shading provides a link between the perceived lighting direction and the perceived object shape (convex or concave), which is not the case for the interpretation of cast shadows.

Few studies on cast shadows have reported a lighting direction assumption. Kersten, Knill, Mamassian, and Bulthoff (1996) showed that a cast shadow moving to a below-right location induced perceived object motion more than one moving to an above-left location. Although their study used a tilted motion path, the results might simply reflect the light-from-above assumption. Koizumi, Ito, Sunaga, and Ogawa (2017) demonstrated the existence of a below-shadow bias (i.e., the light-from-above assumption) using ambiguous cast-shadow stimuli in which disks and shadows were vertically aligned. However, as they did not include obliquely aligned stimuli, they failed to test a possible light-from-above-left assumption for the cast-shadow interpretation.

The light-from-above assumption in the shape-from-shading paradigm can be explained by the position of the sun, which is usually above the observer’s head during the day (Ramachandran, 1988b). However, this assumption can sometimes be inappropriate when interpreting a cast shadow. For example, when an observer stands with his or her back to the sun, the cast shadow of a facing object on the ground arises at the farther side of it (i.e., above, in the visual field). Thus, the assumption that the cast-shadow position is below the object is not always valid. Another important difference that affects shading and shadow interpretations is that a cast shadow can arise at a spatial position distant from the object. This raises a correspondence (or ownership) problem between an object and its shadow. In the shape-from-shading paradigm, the perceived convex–concave distinction of an ambiguous shape is a clue to the assumed lighting direction. However, in cast-shadow studies, ambiguity in ownership of cast shadows could be used to test the perceived lighting direction. Although both shading and a cast shadow can indicate the light-source direction, it is unknown whether the preferred lighting directions of the two are the same. In this study, Experiment 1 measured a cast-shadow bias in various orientations to determine the assumed lighting direction that produced the largest cast-shadow bias.

Shape-from-shading studies have also examined the frame of reference for the interpretation of shading information and have distinguished between head-centric and gravitational frames of reference (Howard et al., 1990; Kleffner & Ramachandran, 1992; Ramachandran, 1988a, 1988b). Observers in the aforementioned studies viewed stimuli with an upright or 90° tilted head orientation. The findings consistently indicated that the *above* in the light-from-above assumption is based on a head-centric, not a gravitational, reference frame, at least for adult observers. Yonas, Kuskowski, and Sternfels (1979) found that between the ages of 3 and 8 years, depth perception based on a shading orientation relative to the environment and the light-source position improved, although a head-centric shading orientation was effective for all age groups. Jenkin, Jenkin, Dyde, and Harris (2004) showed that the *up* was predicted by the weighted vector sum of three frames: visual, gravity, and body-defined frames. In Experiment 2, we measured the shadow bias in a head-centric frame with different head (body) orientations by varying adult observers’ viewing postures. To the best of our knowledge, this is the first study on this topic.

## Experiment 1

### Methods

#### Subjects

Eleven students (mean age, 22.1 years) with normal or corrected-to-normal visual acuity participated in the experiment. All were native speakers of Japanese and educated in Japanese school systems. They were naïve to the purpose of the experiments. This study was approved by the local ethics committee of Kyushu University. Written informed consent was obtained prior to the study.

#### Apparatus and stimuli

The stimuli were generated by a computer (MacBook Pro, Apple) and presented on a 24-in. liquid crystal display (Eizo, FORIS FS2332). The screen was treated as a 1,920 (h) × 1,080 (v) pixel matrix. The stimuli were observed within a 32 cm diameter circular window that was constructed with black cardboard and attached to the screen. The observers viewed the stimuli with one eye from a viewing distance of 60 cm. Viewing was through a black cardboard enclosure to eliminate any visual input other than the stimuli. The experiment was conducted in a darkened room.

**Figure 1.**
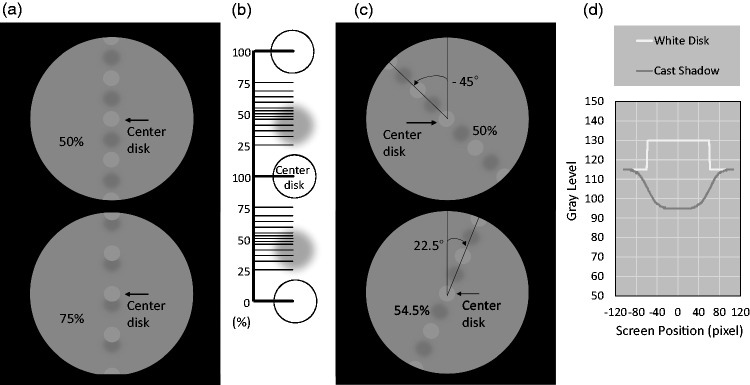
Stimulus configuration. (a) Sample stimulus displays in the vertical (0°) alignment conditions. (b) Shadow positions. In each trial, cast shadows were presented at one of 13 positions shown as thin horizontal lines (25.0%–75.0%). Note that cast shadows were presented on both sides of the center disk. The upper figure in (c) indicates cast shadows on the physically midway position (50%) on the −45° tilted axis. The lower figure in (c) indicates cast shadows on the 54.5% position on the 22.5° tilted axis. (d) Gray-level profiles of a white disk and a cast shadow.^2^

The stimuli were aligned gray disks with pseudo cast shadows positioned between them. Figure 1 shows examples of the stimuli. Five disks (3.8 cd/m^2^) and four pseudo cast shadows (2.0 cd/m^2^) were presented on a gray background (2.9 cd/m^2^).^1^ The Michelson contrast was 31%. The size of the disks and pseudo cast shadows was 2.8° and about 3.0° in diameter, respectively (the edges of the shadow images were blurred on Power Point, see Figure 1(d)). The center-to-center distance of the disks and cast shadows was 6.7°.

We defined a shadow position as the percentage of distance between the center disk and the disk below. When the distance was 50.0% (top panel in Figure 1(a)), the cast shadow was presented in the middle of the disks; that is, the distance between the center disk and the cast shadow above was equal to the distance between the center disk and the shadow below. If there is no directional bias in cast-shadow interpretation, the center disk in Figure 1(a) should be associated with the shadow above or below with the same probability. The bottom panel in Figure 1(a) shows the stimulus display with shadows at the 75% distance. In this case, the center disk and the shadow below may be strongly associated. Similarly, when shadows are at the 25% distance, the center disk may be associated with the shadow above. We varied the percentage distance of the shadows using 13 positions (25.0, 31.8, 36.4, 40.9, 45.5, 47.7, 50.0, 52.3, 54.5, 59.1, 63.6, 68.2, and 75%, shown as thin horizontal lines in Figure 1(b)) to determine the percentage distance of cast shadows when the association rates were equal between the shadows above and below (i.e., the strength of association was balanced for the two shadows). For example, if the association is balanced at the 45% distance in the vertically aligned condition, this indicates that the shadow below is more strongly associated with the center disk than the shadow above. We defined this as a below-shadow bias of 5%. The shadow bias direction indicates an assumption that light is coming from the opposite direction (Koizumi et al., 2017).

We measured the amount of directional bias in the disk–shadow-matching task, changing the orientation of stimulus alignment (see Figure 1(c)). Orientations from −90° to 67.5° in 22.5° steps were tested. 0° indicates the vertical orientation. Negative numbers indicate a counterclockwise tilt, and positive numbers indicate a clockwise tilt.

#### Procedure

Before the experimental sessions, an experimenter explained to participants that the displays were the simulated images of disks and their shadows cast on a vertical wall and instructed the participants to interpret the images as such.

A trial sequence consisted of presentation of a fixation cross, a 500 ms stimulus presentation, and presentation of one green and one blue arrow. Each arrow indicated one of the shadows adjacent to the center disk. The observers’ task was to report verbally (by color name) which arrow indicated the perceptually matched cast shadow.

There were eight alignment orientation conditions. On each orientation axis, the bias of shadow-matching direction was measured using the constant stimuli method; 390 trials (13 shadow positions × 30 repetitions) were performed in a random order for each of the eight orientation conditions. A logistic function was fitted to calculate the percentage distance at which the center disk and shadows in the two opposite directions on an axis were associated with an equal probability.

### Results and Discussion

**Figure 2.**
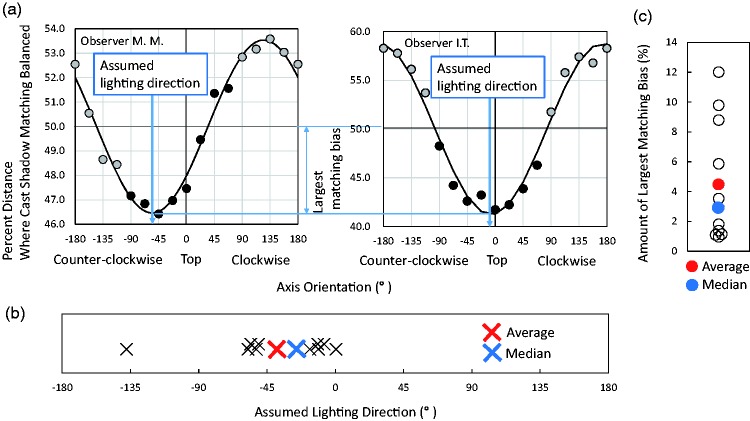
Results of Experiment 1. (a) Sample results from observers M. M. and I. T. Black circles indicate the shadow percentage distance at which either-side shadows were equally associated with the center disk. As each measured axis shares two opposite orientations, the measured data (black circles) were flipped and plotted as data from the opposite orientation (gray circles). After fitting a sine curve to the data, the trough orientation at which the bias was the largest was calculated for each observer (e.g., −55.4° or −6.8° for observer MM or IT, respectively). These orientations correspond to the assumed lighting directions. (b) The trough orientation for 11 observers. (c) The amount of the matching bias in the trough orientation for 11 observers.

Figure 2(a) shows sample results from observers M. M. and I. T. The horizontal axis indicates the angle from the vertical orientation. The vertical axis indicates the balanced cast-shadow percentage distance at which cast shadows on both sides were chosen with equal frequency. The black circles indicate the data obtained. For observer M. M., the balanced percentage distance was lowest at −45°. This indicates that the assumed lighting direction for this observer was above-left.

It is important to note that the stimulus alignment at the −90° orientation was identical to that at 90°. Similarly, the alignment at −22.5° was identical to that at 157.5°. Thus, our stimulus conditions covered 360° in 22.5° steps, pairing the opposite directions on each alignment axis (i.e., sharing the same measurement axis). An orientation axis of −90°, −67.5°, −45°, −22.5°, 0°, 22.5°, 45°, or 67.5° corresponded to an orientation axis of 90°, 112.5°, 135°, 157.5°, 180°, −157.5°, −135°, or −112.5°, respectively. For example, in the −22.5° tilted orientation condition, when the perceptually balanced shadow distance was 46%, the amount of bias was 4%. Then, the amount of bias in the 157.5° tilted orientation condition was automatically determined as −4%. Therefore, the data for the latter orientations were plotted again (gray circles in Figure 2(a)) as reversed data acquired from the former orientation conditions (black circles).

To obtain a more precise analysis, we followed Mamassian and Goutcher (2001) and Gerardin et al. (2010) and fitted a sine curve to the data from each observer (see Figure 2(a)). We used a sine function because the data periodicity had to be 360°, and a phase difference of 180° meant reversing the amount of bias in our measurement method. In addition, the parameters were convenient for further analyses; that is, the trough orientation indicates the orientation that exhibits the largest shadow-matching bias (i.e., showing the preferred lighting direction), and the amplitude corresponds to the amount of matching bias. We calculated the trough orientation and the amplitude for each of the 11 participants. The results are plotted in Figure 2(b) and (c). We statistically analyzed whether the preferred lighting direction was different from 0° (the above). The average of the preferred lighting directions was 38.27° left from the top with a standard error of 11.87°. The median was 25.07° left from the top. These shifts were significant, one-sample *t* test: *t*(10) = −3.223, *p* = .009, Cohen’s *d* = 0.972; one-sample Wilcoxon signed-rank test: *z* = −2.845, *p* = .004, *r* = .858. This orientation value (i.e., 38.27° or 25.07° to the left) is close to those reported for shape-from-shading studies (Andrews et al., 2013; Mamassian & Goutcher, 2001; Sun & Perona, 1998).

## Experiment 2

Experiment 1 showed that the light-from-above assumption was used in the interpretation of cast shadows (except for one observer) and that the assumed lighting direction that caused the strongest directional bias in shadow matching may be tilted to the left. Experiment 2 investigated whether the *top* (or *above*) in the cast-shadow interpretation was defined according to the head-centric frame or the gravitational frame of reference.

### Methods

Thirteen observers (mean age 22.4 years) with normal or corrected-to-normal visual acuity participated in the experiment. All the observers in Experiment 2 were new. Written informed consent was obtained prior to the study. Subjects were naïve to the purpose of the experiments.

**Figure 3.**
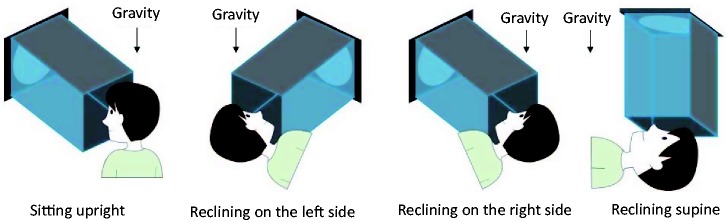
Body posture conditions.

The same apparatus was used to display stimuli as in Experiment 1. To isolate the head-centric frame from the gravitational frame of reference, participants viewed the display in different body postures (thus, different head orientations). The four body posture conditions were sitting upright, reclining on the left side, reclining on the right side, or reclining supine (see Figure 3). A sofa bed and pillow were used to help subjects retain their posture. The stimulus alignment orientations tested for each posture condition were vertical (0°) and horizontal (−90°) in a head-centric frame of reference. The spatial relationship between a participant and the display was kept constant. The four posture conditions were conducted in different blocks. The order of the posture conditions was randomized for each observer. The other procedures were the same as in Experiment 1, except for the presented shadow positions used in the constant stimuli method (i.e., 31.8, 40.9, 43.2, 45.5, 47.7, 50.0, 52.3, 54.5, 56.8, 59.1, and 68.2%).

### Results and Discussion

**Figure 4.**
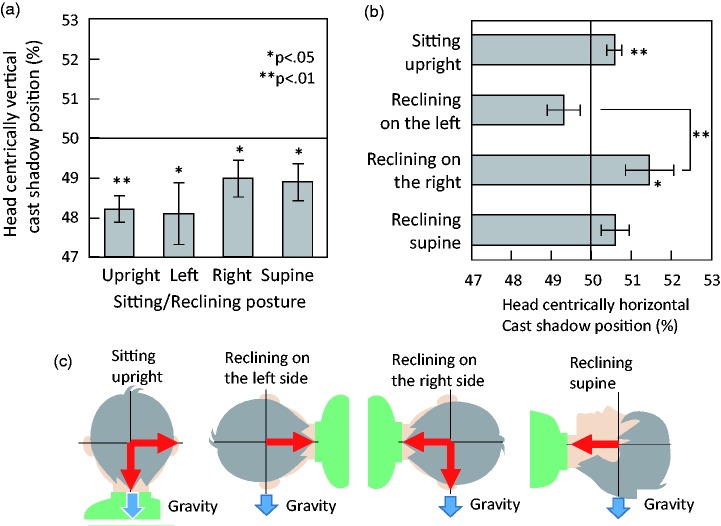
Results. (a) The perceptually balanced position of cast shadows in the head-centric vertical dimension. (b) The perceptually balanced position of cast shadows in the head-centric horizontal dimension. Error bars in (a) and (b) indicate standard errors. (c) The directional shadow biases found in each posture condition, summarizing (a) and (b). Red arrows denote directions of significant biases.

As shown in Figure 4(a), under all body posture conditions, the perceptually balanced shadow distance was significantly below 50% in a head-centric vertical axis, indicating a matching bias toward a head-centric lower shadow, sitting upright: *t*(12) = −5.71, *p* < .001, *d* = 1.58; reclining on the left side: *t*(12) = −2.54, *p* = .026, *d* = 0.70; reclining on the right side: *t*(12) = −2.29, *p* = .041, *d* = 0.63; reclining supine: *t*(12) = −2.47, *p* = .030, *d* = 0.68. A one-way analysis of variance (ANOVA) revealed that the effect of posture was not significant, *F*(3, 36) = 2.06, *p* = 0.12, η*_p_*^2 ^= 0.15. These results demonstrate the robustness of the light-from-above assumption with a head-centric frame of reference.

We found a small amount of matching bias toward a head-centric right shadow in the sitting upright condition, *t*(12) = 3.19, *p* = .008, *d* = 0.88, as shown in Figure 4(b). Experiment 1 showed that the assumed lighting direction in cast-shadow interpretation was 38.27° (on average) tilted to the left. Thus, the small right-shadow bias may reflect the horizontal component of the assumed lighting direction. However, the average directional matching biases in the upright sitting condition were 1.8% below and 0.6% right. This may correspond to the lighting direction of 18.4° left from above, assuming that the upper-left directional bias equals the vector sum of the horizontal and vertical biases. This value seems to be a little different from that in Experiment 1. We think that the horizontal matching bias is less effective when it is decomposed and solely measured.

In the reclining-on-the-left-side condition, head-centric *right* is *above* in the gravitational frame. Thus, there may be a conflict between the gravitational below-shadow bias and the head-centric right-shadow bias. This may have cancelled out the two effects, *t*(12) = −1.77, *p* = .100, *d* = 0.49 (Figure 4(b) and 4(c)).

Conversely, in the reclining-on-the-right-side condition, the head-centric *right* corresponded to the gravitational *below*. Thus, the possible shadow bias directions (i.e., the gravitational below-shadow bias and the head-centric right-shadow bias) may be consistent, resulting in a significant directional bias to the head-centric right, *t*(12) = 2.51, *p* = .027, *d* = 0.70 (see Figure 4(b) and (c)).

There should be no effect of gravitation in the supine condition, because the gravitational, head-centric-horizontal, and head-centric-vertical axes were mutually perpendicular. Although the light-from-above assumption in the head-centric frame was robust here, the right-shadow bias was weakened, *t*(12) = 1.74, *p* = .110, *d* = 0.48. This could be because the left–right axis is better defined when gravitation is involved in spatial cognition. A one-way ANOVA revealed a significant effect of posture, *F*(3, 36) = 4.83, *p* = .006, η*_p_*^2 ^= 0.29. The difference between the reclining-on-the-left and the reclining-on-the-right posture conditions was significant (Ryan’s method, *p* < .01).

One could argue that making the head-centric and gravitational axes perpendicular by looking down the facing-up display is a more ecologically valid condition than the present supine condition. However, we thought that some observers would find it difficult to retain the body posture of lying on the stomach during a block. To reduce observer load, we used a supine posture. It is possible that this nonecological condition might enhance or reduce the effect of the head-centric or gravitational frame. The loss of right-shadow bias in the supine condition might indicate this influence. The effect of ecological validity on cast-shadow matching should be tested in a further experiment.

In short, the light-from-above assumption in the head-centric frame of reference is robust irrespective of body posture (head orientation) when observers interpret ambiguous cast shadows. A small right-shadow bias was also found. This may reflect a horizontal component of the tilted axis of the light-from-above assumption. There may be a possible interaction between head-centric and gravitational frames in cast-shadow matching.

## General Discussion

The results of the two experiments demonstrated some directional bias characteristics of the cast-shadow-matching process. The first characteristic is the presence of a light-from-above-left assumption, although there were large individual differences in preferred lighting direction (as shown in Figure 2). The second is that the light-from-above-left assumption is mainly based on the head-centric frame of reference. The third is the possibility of interaction between the head-centric and gravitational frames of reference. These characteristics may be common to both shading and cast-shadow processing and suggest that the lighting assumption is shared by both processes.

The mechanism underlying the tilt of assumed lighting direction from the top remains to be elucidated in both the shape-from-shading and cast-shadow-matching paradigms. Sun and Perona (1998) demonstrated that right-handers showed stronger leftward bias in lighting direction than left-handers, although other researchers have failed to demonstrate such a difference (Adams, 2007; Andrews et al., 2013; Mamassian & Goutcher, 2001). In Experiment 1, we were unable to test the effect of handedness because only one participant was left-handed. The effect of handedness on shadow matching remains an open question. It is possible that our findings were influenced by cultural effects (Andrews et al., 2013). All our participants were Japanese. In Japan, there are two types of books or notebooks: those that can be read or written in from right to left and those that can be read or written in from left to right. Thus, the leftward bias for first-language Japanese participants could be weaker than that for first-language English participants. However, we found that the leftward bias found in our shadow-matching experiment was as great as that of the first-language English participants in a study on shape-from-shading displays (Andrews et al., 2013). Classrooms in most Japanese elementary and junior high schools have windows on the left (south side), facing the teacher’s desk (west side), to obtain natural lighting from outside. This lighting direction is intended to be convenient for right-handed people and ensures that the right hand does not cast a shadow onto books or notebooks. This could constitute a leftward bias of lighting as a cultural practice. However, we could not find a Japanese study that precisely estimated the assumed lighting direction in relation to shape from shading or cast-shadow matching. Sawada and Kaneko (2003) confirmed the light-from-above bias in the shape-from-shading paradigm. Inoue and Watanabe (2005) showed that perceived depth was greater when a *highlight* was positioned at an upper location inside a circular form than when it was positioned at a lower location. Although both studies demonstrated the light-from-above assumption, the assumed lighting direction was not precisely assessed. The effects of cultural factors on assumed lighting direction is still unclear.

The results of Experiment 2 suggest the effect of gravity on the interpretation of cast shadows, as Jenkin et al. (2004) have shown in the shape-from-shading paradigm. As Koizumi et al. (2017) have shown, shape from shading may be a local process, whereas cast-shadow interpretation should collect information from a scene because cast shadows are sometimes distant from their objects. Thus, cast-shadow interpretation may depend more on the visual frame of reference. This possibility should be investigated in the future.

In this study, we investigated preferred lighting direction only in horizontal and vertical dimensions, not including the front–rear dimension. However, it would be feasible to position a light source in three dimensions. The light source was always assumed to be positioned on the viewer side of the screen in our experiments. This is a problem partly shared with previous studies conducted within the shape-from-shading paradigm. In this study, cast shadows could also change position according to the change in distance between the light source and the disk and also the distance between the disk and the wall. Our results indicated some preference for the light-source direction. One of the next challenges is to incorporate the depth dimension into the present paradigm.

## Authors’ Note

Part of the results was presented at the *31st International Congress of Psychology* (ICP2016), held in Yokohama, Japan. Tomomi Koizumi is now affiliated to Benesse Style Care Co.,Ltd (not an academic job). Masaki Ogawa is currently affiliated to Faculty of Engineering, Mie University.

## Notes

1 The luminances were set rather low to reduce the visual fatigue of observers, who viewed the display with one eye through a dark enclosure for a total of 4 hours. Before conducting Experiment 1, we conducted an experiment similar to Experiment 1 except that the directional conditions were halved. The results confirmed that these luminance conditions were sufficient to measure the effect that we planned to investigate. However, it is possible that better luminance and contrast combination conditions for this experiment exist.

2 *Cast shadows* were dark-gray disks that were blurred using Microsoft PowerPoint 2016 (*soft edges* function, *size* = 20 point). The size of the dark-gray disk was set at 170%, larger than the white disk, to correct apparent size change caused by this processing. We confirmed that the produced gray-level profile was consistent with that produced using Gaussian filtering with ImageJ (NIH).
